# Accelerator mass spectrometry (AMS) in plutonium analysis

**DOI:** 10.1007/s10967-013-2448-4

**Published:** 2013-03-02

**Authors:** Dagmara I. Strumińska-Parulska

**Affiliations:** Faculty of Chemistry, Analytics and Environmental Radiochemistry Chair, University of Gdańsk, Sobieskiego 18/19, 80-952 Gdańsk, Poland

**Keywords:** Plutonium, ^240^Pu/^239^Pu atomic ratio, AMS accelerator mass spectrometry, Chernobyl accident, Atmospheric fallout, Fish

## Abstract

The paper summarizes the results of the ^240^Pu/^239^Pu atomic ratio studies in atmospheric fallout samples collected in 1986 over Gdynia (Poland) as well as three Baltic fish species collected in 1997 using the accelerator mass spectrometry. A new generation of AMS has been developed during last years and this method is an efficient and good technique to measure long-lived radioisotopes in the environment and provides the most accurate determination of the atomic ratios between ^240^Pu and ^239^Pu. The nuclide compositions of plutonium in filter samples correspond to their means of production. AMS measurements of atmospheric fallout collected in April showed sufficient increase of the ^240^Pu/^239^Pu atomic ratio from 0.28 from March to 0.47. Also such high increase of ^240^Pu/^239^Pu atomic ratio, close to reactor core ^240^Pu/^239^Pu atomic ratio, was observed in September and equaled 0.47.

## Introduction

The measurement of isotopic ratios such as ^240^Pu/^239^Pu can provide information on the source of contamination. Most of the environmental studies demand low detection limits for the alpha-emitter ^239^Pu (*T*_1/2_ = 24 110 a, *E* = 5,155.5 keV (73.3 %), 5,142.8 keV (15 %), 5,104.7 keV (10 %)), the fissile and the major isotope. In addition to ^239^Pu, ^240^Pu (*T*_1/2_ = 6,564 a; *E* = 5,168.2 keV (73 %), 5,123.7 keV (26 %)), the second major isotope produced via neutron capture of ^239^Pu, deserves special attention, as the ^240^Pu/^239^Pu ratio offers unambiguous information about the plutonium source [1, 2]. However, the measurement of anthropogenic actinides in the environment is difficult, as they are present in the very low concentrations and often have long half-lives, which makes measurements by means of radioactive decay counting difficult or even impossible [2–5]. Alpha spectrometry is commonly used for determination of actinides in environmental samples. This technique requires the radio-tracers and long-lasting as well as laborious work. However, it gives excellent detection limits. Alpha spectrometers typically require 200 keV peak to peak separation for baseline resolution between successive alpha emission of different isotopes [6]. Even with the lowest 18 keV resolution of the typical PIPS detectors used during alpha spectrometry measurements, it is difficult to determine the abundance of ^239^Pu and ^240^Pu isotopes in samples because the five primary alpha emissions for the two isotopes lie within a range of 65 keV, with the lowest difference at 13 keV.

Accelerator mass spectrometry (AMS) is a modern analytical technique which allows the long-living radioactive radionuclides to be measured in environmental samples [4, 7–10]. New generation AMS enables the heaviest elements (actinides) to be detected and the isotopic ratios between them to be estimated. Mass-spectrometric methods (e.g. AMS, TIMS, ICP-MS) do give information on the ^240^Pu/^239^Pu ratio, and potentially have higher sensitivity than α-particle counting, with values as low as ~1 fg having been reported, but are sensitive to molecular interferences, i.e. ^238^UH, ^208^Pb, ^31^P, etc. could interfere with measurement of ^239^Pu [11, 12]. AMS is characterized by low measurement background and high detection efficiency; separation of isotopes is possible, but (atomic or molecular) isobars can not be resolved [3, 13, 14].

In the case of plutonium it is possible to measure femtograms (10^−15^ g) of this element and calculate the isotopic ratios of ^238^Pu/^240^Pu and ^240^Pu/^239^Pu [3, 7, 8, 10, 15]. The main advantage of AMS compared to conventional MS (TIMS, ICP-MS) is the complete destruction of molecular isobars (e.g. ^238^UH for ^239^Pu) by stripping to high positive charge states in the terminal of the tandem accelerator [3, 13, 14]. AMS can minimize the sample size requirements due to its low detection limit of below 10^6^ atom of ^239^Pu. Further, purification of plutonium leached from the discs after semiconductor alpha spectrometry is not necessary. However, AMS operation is complex and a well experienced team is required. Also, the analysis is quite expensive [16]. Summarizing, the AMS has very short analysis times, high sensitivity and enables the measurement of the distinction of ^239^Pu and ^240^Pu and sometimes ^241^Pu [4, 17]. In spite of these advantages, it is not yet possible to determine ^238^Pu due to the interferences of ^238^U [18].

The Baltic Sea is a small shelf sea, very sensitive to human impact and marine plants and animals accumulate radionuclides from the aquatic environment [19–24]. The main source of plutonium in the Baltic Sea and the Poland territory, before the Chernobyl accident, was global fallout from the nuclear weapons testing. Other sources of plutonium, e.g. releases from the spent fuel facilities at Sellafield (UK) and Cap de la Hague (France), are less important. Since 26 April 1986 there has been a new source of plutonium—the Chernobyl accident—which should be taken into consideration in environmental risk assessments [19]. Different sources often exhibit characteristic Pu isotope ratios, and these ratios are known to vary with factors like nuclear fuel burn-up, reactor type and operating history, neutron flux and weapon yield. Hence, these ratios can be used to identify the origin of contamination, calculate inventories [25].

**Fig. 1:**
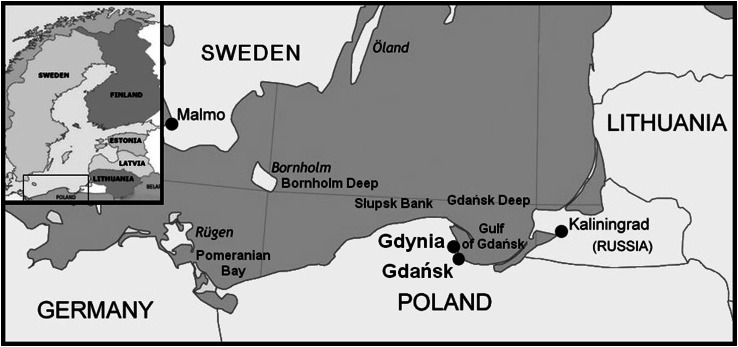
The southern Baltic Sea

This work presents the results of the ^240^Pu/^239^Pu isotope ratios determination in air dust samples collected over Gdynia (Poland) and three Baltic fish species using AMS technique (Fig. 1).

## Materials and methods

The atmospheric air dust samples were collected monthly in 1986 over Gdynia (northern Poland) while three species of fish: flounder, herring and cod from the Gulf of Gdańsk (the southern Baltic Sea) were caught in 1997 (Fig. 1). The whole fish were measured and weighed. Organs and tissues were dissected from fresh fish immediately after their delivery to the laboratory; the following tissues and organs were isolated: muscles, skeleton, skin, gills, stomach, intestine, liver, gonads and rest [22, 24]. All samples preparations were carried out in clean, low-level analytical laboratories. The pre-weighted filters with air dust samples as well as isolated fish organs and tissues were weighted and digested using 65 % HNO_3_ with a ^242^Pu (5 mBq) spikes added as a yield tracers before the radiochemical analysis. The analysis of plutonium treated as follows: samples mineralization in nitric acid and additionally in HF in the case of air dust samples, separation and purification on ion exchange resins, electrolysis on a steel disc and determination of plutonium ^238^Pu and ^239+240^Pu isotopes activities by alpha spectrometry [19, 20, 22, 24, 26]. After radiochemical preparation, the activities of ^238^Pu and ^239+240^Pu radionuclides were measured separately by alpha spectrometry (Alpha Analyst S470, Canberra Packard, USA) equipped with 12 PIPS detectors (300 or 450 mm^2^ area each, FWHM = 18 keV). The accuracy and the precision of the plutonium analysis were satisfactory, i.e., precision less than 7 % (1.5–6.4 %), as estimated by analysis of the International Atomic Energy Agency standard materials (IAEA-367, IAEA-384, IAEA-414). The chemical yield of plutonium analysis varied from 60 to 90 %. The results are also given with standard deviation (SD) calculated for 95 % confidence intervals. The detection limits were calculated at 0.05 mBq for ^239+240^Pu [27].

**Fig. 2:**
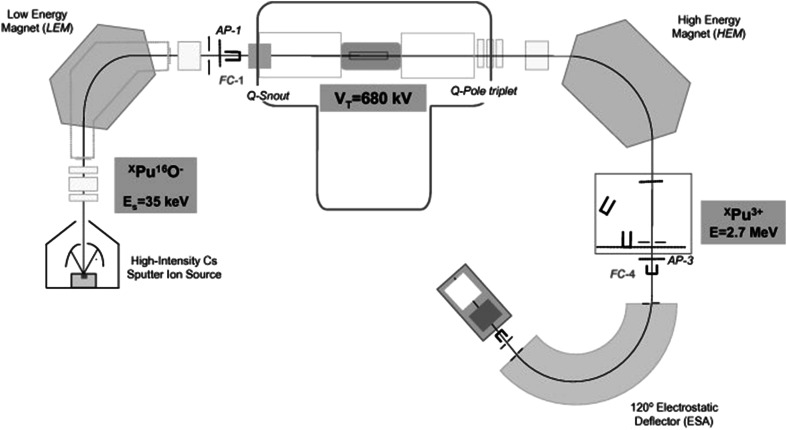
Main components of the 1 MV AMS system at the CNA (Spain)

The ^240^Pu/^239^Pu atomic ratios were measured in 2007 using 1 MV compact AMS system located at Centro Nacional de Aceleradores (CNA) (Seville, Spain). All sources of air dust and fish samples previously separated, purified and measured ^238^Pu and ^239+240^Pu using the alpha spectrometer, were prepared for AMS measurements. The samples (alpha spectrometry targets) were dissolved in 2 M HNO_3_ and the plutonium fractions were transferred to the solution. After isolating the sample from the disc, 1 mg of iron (i.e. as Fe(NO_3_)_3_) was added and then the solution was evaporated in the PTFE tube. The iron was joined with the plutonium and used as the carrier. After evaporating, the Pu/Fe matrix was transferred to the ceramic crucible and baked in 800 °C for 3 h, to convert the nitrate components to oxides. Further 2 mg of aluminum powder was added, to give the samples indispensable electric and thermal conductivity. After that the sample was pressed into the aluminum holder with thin Cu wire and the Al/Cu target cathode was prepared [2, 10, 25, 28, 29]. AMS system at CNA is a 1 MV Tandetron^®^ system is equipped with a carousel for 200 samples and the accelerator. Argon gas is used as a stripper. Finally, the ions are counted with a two-anode gas ionization detector provided with a 75 nm thick 8 × 8 mm^2^ silicon nitride window (Fig. 2; [2]). The mass or energy resolution of the magnets are 680 and 1,300 mm/(Δ*M*(*E*)/*M*(*E*)), respectively. At the image of the ESA, whose energy resolution is 1,300 mm/(Δ*E*/*E*), a pair of horizontal slits helps as well to control the final energy of the particles arriving at the detector. The software of AMS, designed to support the injection of different masses into the detector by changing the tuning of the system sequentially, allows us to perform automatic measurement of plutonium isotopes. Usually, pulses of 15 s are dedicated to ^239^Pu, of 30 s to ^240^Pu and of 5 s to ^242^Pu. The final results are the ^239,240^Pu/^242^Pu atomic ratios. ^242^Pu is used as the normalization isotope for the measurements and as the radiochemical tracer (previously used in alpha spectrometry analysis) for the chemical procedure in the case of processed environmental samples. A single measurement is completed when this sequence is repeated 5 times, and a real sample analysis comprises usually between five and seven single measurements alternated with standards and blanks. The typical instrumental error of the final ratios is about 1.5 % (standard deviation of the mean). The AMS technique was applied to three reference materials IAEA-381, IAEA-Soil-6 and IAEA-375 and the accuracy and precision were estimated. The IAEA certifies the ^239+240^Pu activity concentration for all of them, but the ^240^Pu/^239^Pu atomic ratio only for the sample 381 while for the IAEA-Soil-6 ^240^Pu/^239^Pu atomic ratios were referred to the results given by Muramatsu et al. [30]. The accuracy as well as the precision were satisfactory, estimated at less than 5 % for ^240^Pu/^239^Pu measurements and less than 2 % for ^239+240^Pu measurements [2, 29]. The detection limits were achieved at ∼10^6^ atoms for each isotope [10].

However, according to our fish samples, the ^239+240^Pu contents were too low to measure ^240^Pu/^239^Pu atomic ratios in most of analyzed organs and tissues.

## Results and discussion

Statistical analysis of the ^239+240^Pu concentrations as well as the ^240^Pu/^239^Pu atomic ratio values in analyzed samples showed that the differences between them were statistically significant, in spite of their uncertainties. The plutonium nuclide compositions in the filter samples corresponded to their means of release into the environment. The measurements of the atmospheric fallout samples collected monthly throughout 1986 allowed the Chernobyl peak effect on Gdynia (Poland) to be observed (Table 1). From January to March 1986 we also observed quite high ^240^Pu/^239^Pu atomic ratios (from 0.24 to 0.36) when compared to the global stratospheric fallout ^240^Pu/^239^Pu atomic ratios (0.18) [31]. The values of ^240^Pu/^239^Pu atomic ratios given for the global stratospheric fallout were 0.176 ± 0.014 and 0.180 ± 0.014, weapon-grade Pu generally presents a very low ^240^Pu/^239^Pu atomic ratio varying from 0.02 to 0.06 and those from close-in tropospheric fallout from the Pacific Proving Grounds (PPG) in the Marshall Islands was 0.33–0.36 [2, 4, 31–36]. According to the Polish conditions (East Europe) the average value for the global atmospheric fallout (^240^Pu/^239^Pu atomic ratio at 0.18) is more probable. Air filter data measured from January to March 1986 should correspond with typical values for material affected only by the global fallout. One of the reasons for the enhanced ^240^Pu/^239^Pu atomic ratio in the air dust over Poland could, for example, have been the Chernobyl-1 accident in September 1982. This time the part of the core reactor was released into the industrial zone and city of Pripyat, causing irradiation of the surroundings [37]. The other, more probable reason is the impact of aerosols from the North Sea inflows. The atomic ratios of ^240^Pu/^239^Pu from the western European nuclear fuel reprocessing facilities were 0.34 ± 0.03 in the effluent from Cap de la Hague and they were reported over Poland [38, 39]. Although other accidents should also be recalled (e.g. Kyshtym 1957, Leningrad 1975) the global atmospheric fallout had the main impact on the ^240^Pu/^239^Pu atomic ratios [40, 41]. The differences and significant decrease of ^240^Pu/^239^Pu atomic ratios as well as ^239+240^Pu concentrations in January and February 1986 can be explained by very low precipitation in February 1986 caused by continental high-pressure area from Russia which brought very low temperature and lack of snow–the average monthly precipitation over Gdynia in January, February and March 1986 was calculated at 44, 5 and 22 mm and the average monthly temperature in February was −5.5 °C while in January only −0.1 °C. It could mean lower impact of the western nuclear fuel reprocessing facilities with higher ^240^Pu/^239^Pu atomic ratios. Weather changes as well as higher temperatures in March 1986 brought more precipitation what influenced on higher ^240^Pu/^239^Pu atomic ratio [42].

**Table 1:** ^239+240^Pu concentration and ^240^Pu/^239^Pu atomic ratio in airborne dust collected over Gdynia (Poland) in 1986, calculated for 95 % confidence intervals (2*σ*)

Sample	^239+240^Pu concentration [24] (μBq g^−1^ dw ± SD)	^240^Pu/^239^Pu atomic ratio
January	4.14 ± 0.67	0.36 ± 0.06
February	32.9 ± 3.2	0.23 ± 0.03
March	26.2 ± 3.6	0.29 ± 0.04
April	65,122 ± 723	0.47 ± 0.02
May	662 ± 100	0.38 ± 0.02
June	61.7 ± 25.2	0.41 ± 0.06
July	58.2 ± 12.7	0.49 ± 0.10
September	41.2 ± 13.0	0.47 ± 0.07
November	18.4 ± 5.8	0.44 ± 0.15
December	26.3 ± 5.9	0.37 ± 0.10

Obtained data indicated a significant increase in the ^240^Pu/^239^Pu atomic ratio from 0.29 ± 0.04 in March 1986 to 0.47 ± 0.02 in April 1986 (Table 1). Our value corresponds with the ^240^Pu/^239^Pu atomic ratios calculated for the reactor core (from 0.39 to 0.563) [43, 44]. Such a high ^240^Pu/^239^Pu atomic ratio (0.42) was also observed a few days after the Chernobyl accident in aerosols over Vilnius (Lithuania) [45]. From November 1986, the ^240^Pu/^239^Pu atomic ratios decreased and had returned to similar level as before the accident [19]. In December 1986 the ^240^Pu/^239^Pu atomic ratio was calculated at 0.37 ± 0.10, which is close to the value recorded in January 1986—0.36 ± 0.04. Also ^239+240^Pu concentration from December 1986 (26.3 ± 5.9 μBq g^−1^ dw) was similar to those reported for January and February 1986 (32.9 ± 3.2 and 26.2 ± 3.6 μBq g^−1^ dw respectively). Slightly lower ^240^Pu/^239^Pu atomic ratios were found in forest soil from north-eastern Poland a few years later: Szczerba 0.309 ± 0.011, Plaska 0.348 ± 0.011, Trzcianka 0.308 ± 0.024 [46]. Although ^239+240^Pu concentrations obviously increased, we can clearly expect ^240^Pu/^239^Pu atomic ratios to continue falling achieving the values typical for the global atmospheric fallout with an impact of the nuclear fuel reprocessing facilities. Over Vilnius (Lithuania) this ratio decreased from 0.42 in 1986 to 0.20 in 2002 [45].

The measurements of fish samples allowed obtaining only a few results, while most of them were below the detection limit, probably the ^239+240^Pu content was too low to be measured (Table 2). However, obtained data were much differentiated; the highest ^240^Pu/^239^Pu atomic ratio was found in stomach of pelagic plankton-feeding herring (*Clupea harengus*) (0.44 ± 0.03) and the lowest in liver of pelagic predatory cod (*Gadus morhua*) (0.055 ± 0.016) (Table 2). High ^240^Pu/^239^Pu atomic ratio in herring stomach (0.44 ± 0.03) can be connected to its indirect contact with food and water enriched with the Chernobyl plutonium [23]. Low ^240^Pu/^239^Pu atomic ratio in cod liver (0.055 ± 0.016) indicates the liver is not enriched as the other organs of the analyzed fish. Previous studies showed that the highest amount of plutonium in the long-living predatory cod was mainly in intestine and skeleton. The liver probably reflected the plutonium metabolism and its changes in the environment in less extent in comparison to the other organs and tissues and contained mainly the fraction of weapon-grade plutonium [22].

**Table 2:** ^239+240^Pu concentration and ^240^Pu/^239^Pu atomic ratio in fish from the Gulf of Gdańsk collected in 1997, calculated for 95 % confidence intervals (2*σ*)

Sample	^239+240^Pu concentration [22] (μBq g^−1^ ww ± SD)			^240^Pu/^239^Pu atomic ratio		
	Herring	Cod	Flounder	Herring	Cod	Flounder
Muscles	0.24 ± 0.05	0.29 ± 0.06	0.13 ± 0.02	0.27 ± 0.01	bdl	0.32 ± 0.01
Skeleton	1.01 ± 0.11	0.91 ± 0.20	0.67 ± 0.09	bdl	bdl	bdl
Skin	1.12 ± 0.14	0.54 ± 0.08	0.63 ± 0.10	bdl	bdl	0.26 ± 0.04
Gills	37.73 ± 3.47	6.21 ± 0.78	5.32 ± 0.63	bdl	bdl	bdl
Stomach	2.31 ± 0.42	1.97 ± 0.26	2.03 ± 0.32	0.44 ± 0.03	bdl	bdl
Intestine	4.09 ± 0.39	70.50 ± 3.61	7.11 ± 0.52	bdl	bdl	bdl
Liver	9.36 ± 1.13	0.40 ± 0.09	1.51 ± 0.19	bdl	0.055 ± 0.016	bdl
Gonads	4.52 ± 0.42	4.08 ± 0.70	1.68 ± 0.29	bdl	bdl	bdl
Rest	17.15 ± 1.85	1.12 ± 0.23	3.79 ± 0.61	bdl	bdl	bdl

*bdl* Below detection limit

Our previous studies showed the plutonium analyzed in fish skin came mainly from the water as adsorbed by mucus; 66 % of total ^239+240^Pu in skin was connected to mucus, while only 34 % was inbuilt in the true skin and the skales [23]. Low ^240^Pu/^239^Pu atomic ratio in skin of benthic flounder (0.26 ± 0.04) could indicate lower amount of the Chernobyl-derived plutonium in bottom water or could indicate mainly the global atmospheric fallout plutonium with increasing impact of the Chernobyl-derived plutonium. However, the study about determination of plutonium in flora and fauna representatives from the Gulf of Gdańsk in 1987–1991 showed low participation of Chernobyl-derived plutonium and its deposition in sediments (a few percents) and Bivalvia (10 %). Chernobyl plutonium in phytobenthos and fish was not observed at that time [19]. Our later studies carried out in 1997, showed bigger amount of the Chernobyl-derived plutonium in fish from the Gulf of Gdańsk. The highest amounts of the Chernobyl plutonium were found in organs and tissues of pelagic herring and cod (10–80 %) while the lowest in benthic flounder (0–50 %) [22]. Despite all these years from the Chernobyl accident its radiological effects could be still observed in the environment of the southern Baltic caused by the effect of late inflow of plutonium to Baltic Sea increasing the impact of the global atmospheric fallout as well as the nuclear fuel reprocessing facilities.

## Conclusions

We estimated the ^240^Pu/^239^Pu atomic ratios in the air dust as well as Baltic fish and obtained results were much differentiated. The nuclide compositions of plutonium in the filter samples correspond to their means of production. AMS measurements of atmospheric fallout samples collected monthly throughout 1986 allowed the Chernobyl peak effect on Gdynia (Poland) to be observed. Obtained data indicated a significant increase in the ^240^Pu/^239^Pu atomic ratio from 0.29 ± 0.04 in March 1986 to 0.47 ± 0.02 in April 1986 what corresponds with the ^240^Pu/^239^Pu atomic ratios calculated for the reactor core (from 0.39 to 0.563). Further studies showed in November 1986 the ^240^Pu/^239^Pu atomic ratio decreased. According to fish samples most of the analyzed ^240^Pu/^239^Pu atomic ratios were below the detection limit, probably due to low total ^239+240^Pu content in the analyzed samples. However, obtained results showed the radiological effects of the Chernobyl accident could be still observed in the environment of the southern Baltic caused by the effect of late inflow of plutonium to Baltic Sea increasing the impact of the global atmospheric fallout as well as the nuclear fuel reprocessing facilities.

A good option for alpha spectrometry complement is accelerator mass spectrometry (AMS). AMS is characterized by low measurement background, high detection efficiency and what is the most useful, needs the same radiochemical method as alpha spectrometry which is fully applicable for the accurate determination of the plutonium in different environmental matrixes.
